# Challenges of implementation of the preventive chemotherapy neglected tropical diseases programme in Ghana

**DOI:** 10.1371/journal.pntd.0011116

**Published:** 2023-02-21

**Authors:** Desmond Dzidzornu Otoo, Ivy Akushika Agbenu, Mary Adebi Nyamekye, Nana Nimo Appiah-Agyekum

**Affiliations:** 1 Department of Public Administration and Health Services Management, University of Ghana Business School, University of Ghana, Accra, Ghana; 2 Department of Health Policy, Planning and Management, School of Public Health, University of Ghana, Accra, Ghana; Al-Jouf University College of Pharmacy, SAUDI ARABIA

## Abstract

**Purpose:**

The Neglected Tropical Diseases programme since its implementation has improved the lives of many in the tropical and sub-tropical areas. Though witnessed many successes, the programme is continually facing challenges thus, preventing the attainment of various objectives. This study seeks to assess the challenges of implementation of the neglected tropical diseases programme in Ghana.

**Design/Methodology/Approach:**

The thematic analysis approach was used to analyze qualitative data collected from 18 key public health managers selected through purposive and snowballing procedures from the national, regional and district levels of Ghana Health Service. Data collection was done through in-depth interviews using semi-structured interview guides in line with the objective of the study.

**Findings:**

The Neglected Tropical Diseases Programme though received funding from external sources, faces multiple challenges which cut across financial, human and capital resources to external control. Specifically, inadequate resources, dwindling volunteerism, poor social mobilization, weak governmental commitment and weak monitoring were major challenges to implementation. These factors work individually and in combination to impede effective implementation. Ensuring state ownership, re-structuring implementation approaches to include top-down and bottom-up approaches and building capacity in monitoring and evaluation are recommended in order to meet the programme objectives and ensure sustainability.

**Originality:**

This study forms part of an original study on Implementation of the NTDs programme in Ghana. Aside the key issues discussed, it presents first-hand information on major implementation challenges that are relevant to researchers, students, practitioners and the general public and will apply widely to vertically implemented programmes in Ghana.

## Author summary

Diseases classified as neglected continue to affect the poorest of people across the world especially in the Sub-Saharan Africa region. Although efforts have been made, several barriers continually exist to delay attainment of programme objectives. This study assessed the challenges of implementation of the neglected tropical diseases programme in Ghana from the major state implementation agency (GHS) at the national, regional and district levels. We found the challenges of implementation that acted independently and synergistically to be inadequate resources, dwindling volunteerism, poor social mobilization, weak governmental commitment and weak monitoring and evaluation.

## Introduction

The world has witnessed several efforts towards the fight against Neglected Tropical Diseases (NTDs) over the past decade. This has resulted in the redirection of resources towards NTDs control by health stakeholders [1–3].

Globally, governments in disease-endemic countries have supported the control of NTDs through programmes that are independent and often vertical with its own planning, funding, drug supply chain, implementation campaign, monitoring, and evaluation [4].

A key process to the survival and growth of the health sector is policy implementation [5]. Effective implementation in order to meet set objectives has positive impacts on the health system. However, there has been the criticism that documents developed by health sector policy makers are often left on shelves with no or poor implementation. Agencies in some instances fail to utilize the precious information in strategic plan documents [6]. Furthermore, claims have it that, organizations in Africa in most instances do not execute strategies according to the plans in the programme and making the strategy effective in the entire country poses more challenges [7].

The NTDs programme in Ghana was established to improve on the capacity of the Ghana Health Service (GHS) to establish an integrated programme capable of delivering interventions to prevent, control, eliminate or eradicate preventive chemotherapy related neglected tropical diseases. The programme broadly includes diseases tackled with preventive chemotherapy. In Ghana, the most prevalent NTDs controlled by preventive chemotherapy include Onchocerciasis, Lymphatic Filariasis (LF), Schistosomiasis and Soil Transmitted Helminths (STH) [8]. The Global Burden of Diseases (GBD) study reported that schistosomiasis affects about one (1) million people and remains the second most prevalent parasitic infection in Ghana after Malaria [9]. LF is reported to be endemic in 13 out of the 16 regions in Ghana. Also, Onchocerciasis is hyperendemic in 29 districts, meso-endemic in 15 districts and hypo-endemic in 91 districts [8].

Interventions adopted for the control and elimination of NTDs in Ghana include Mass Drug Administration (MDA) on annual and bi-annual bases, morbidity management and disability prevention (MMDP), health education, behaviour change communication (BCC) and vector control using a community directed intervention (CDI) strategy. These interventions are integrated into the health system to maximise utility of available resources to achieve the greatest possible impact [8]. The CDI strategy was employed to encourage communities to prioritize their own health and to cover hard-to-reach areas and communities with compromised securities amidst the inadequacy of health personnel in such areas [10].

Since the ambitious goals to eliminate and control NTDs were launched, the programme has faced many challenges during implementation. This has delayed the rate of meeting the objectives of the programme with the existence of significant burden of NTDs across regions in Ghana.

Studies have suggested that efforts directed at improving performance in the health system must address challenges related to implementation [11]. Though it is important to identify implementation challenges, very few studies have studied the challenges that affect the implementation of the NTDs programme at the level of the major state implementer (Ghana Health Service) with majority being evaluations by funding agencies [12]. There is the urgent need to find out the challenges and sources of such challenges and address them in a more holistic way to ensure effective implementation. Thus, this study sought to determine the challenges of implementation of the NTDs programme in Ghana from the view of the state implementers.

## Materials and methods

### Ethics statement

Ethical clearance to conduct the study was obtained from the Ghana Health Service Ethics Review Committee as part of the Implementation of the NTDS programme study with certificate number GHS-ERC 065/11/19. Appropriate permissions were sought from the national, regional and district directors of health before the data collection was commenced. Written informed consent was sought from all participants. All ethical principles in research were upheld during the study.

### Research design

A descriptive qualitative case study approach was adopted with guides adapted from WHO’s methodology for evaluation of NTDs programmes at country level developed in 2008 [13]. Even though the guides were modified, it served as an essential tool in the study. In order to gain deeper understanding, the study focused on key public health managers responsible for the NTDs programme at the national, regional and district levels.

The study was conducted in the Ga West District in the Greater Accra region and Lower Manya Krobo District in the Eastern region and their respective regional health directorates. The national NTDS control programme was also involved in the study in order to attain national perspectives. The districts were selected conveniently after a scoping review considering districts with co-endemicity of two (2) or more Neglected Tropical Diseases, proximity to researchers during the peak of the Covid-19 pandemic due to restrictions placed in some parts of the country and to reflect urban and rural balance. Lymphatic Filariasis, Schistosomiasis and Soil Transmitted Helminthiasis were endemic in Ga West. Schistosomiasis and Soil Transmitted Helminthiasis were endemic in Lower Manya Krobo [14].

Purposive and snowballing sampling approaches were employed in the recruitment of respondents. The heads of disease control at the Greater Accra and Eastern regional health directorates and Lower Manya Krobo and Ga West district health directorates were purposively sampled. A snowballing technique was then used to recruit subsequent respondents responsible for the NTDs programme with the aid of the initial respondents. The heads of disease control referred the researchers to NTDs focal persons who also led researchers to their colleagues working on the NTDs programme. This strategy ensured that we obtained information from people who were directly working with the NTDs programme. A total of 18 respondents were recruited (Table 1). Similar sampling approaches were discussed in a study on the determinants of NTDs programme implementation success [14] and another that mentioned the use of existing chains of command and reporting for data collection to overcome key challenges in recruiting top level implementers [15]. Sampling was done until saturation after the 19th and 20th respondents were recruited.

**Table 1:** Respondents distribution.

Levels	Respondents
National level	Respondent 1 to Respondent 6
Regional level	Respondent 7 to Respondent 10
District level	Respondent 11 to Respondent 18

**Fig 1:**
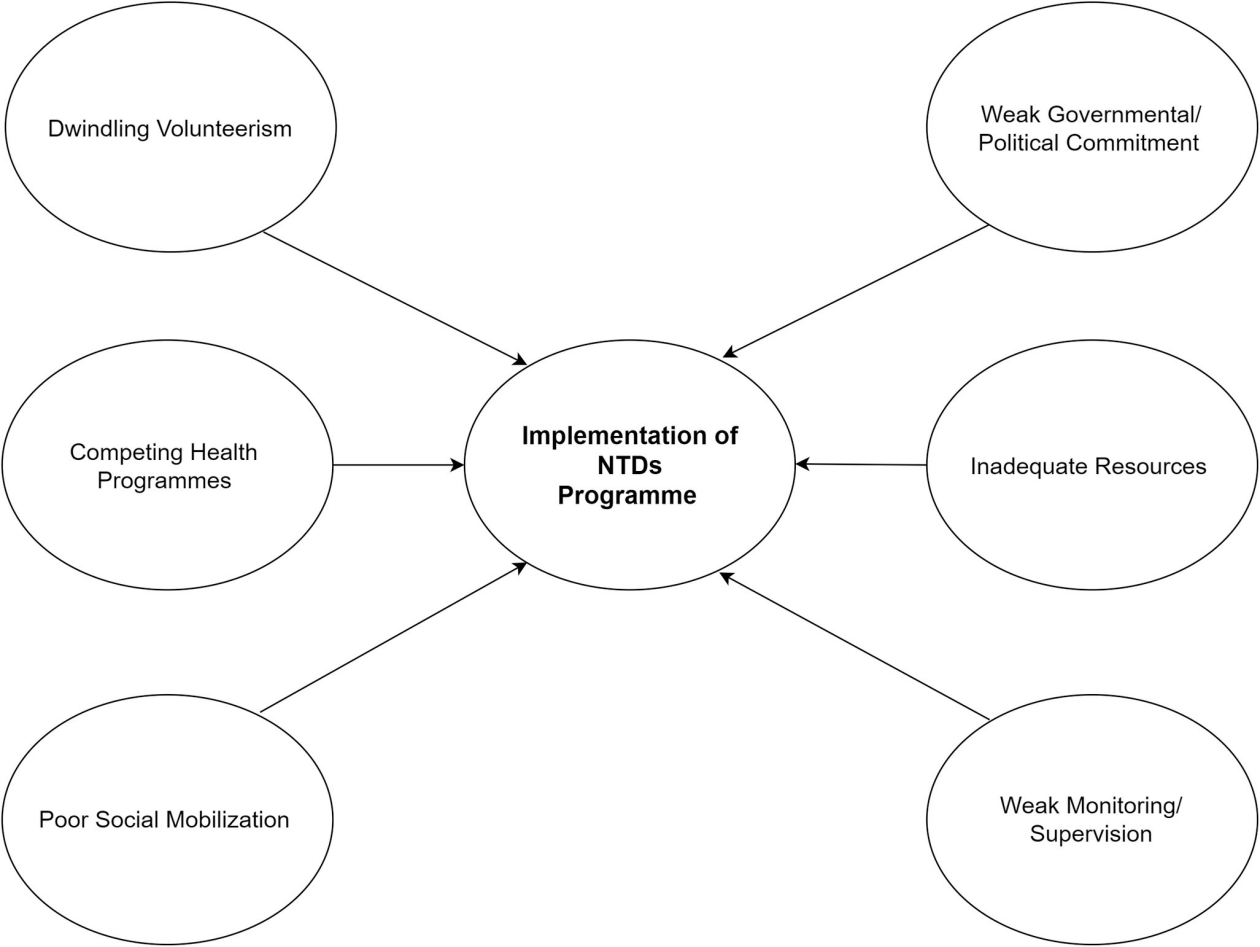
Summary of findings/themes emanating from study.

In-depth interviews were conducted with officers in a total of 4 months period (March to June 2020). Interviews lasted between 60 minutes to 90 minutes using the semi-structured interview guides. The guides were flexible and made use of probes and prompts to enquire experiences of respondents on the implementation challenges. Interviews conducted were recorded with a tape recorder and notepads, transcribed (S1 Text) and shown to respondents for clearance before further analysis were done. Data was analyzed using the thematic analysis approach with the help of NVivo 11. Emergent themes from the analysis (Fig 1) were discussed with relevant literature.

## Results

The study sought to find out the challenges of implementation of the NTDs programme. Findings indicate that inadequate resources dwindling volunteerism, poor social mobilization, competing health programmes, weak governmental/political and weak monitoring and supervision were major challenges hindering successful implementation (Fig 1).

### Inadequate resources

Respondents revealed that though the programme receives funding from key external partners and some local agencies, the inadequacy of funds and other major resources have generally been a challenge to effective implementation. It was added that in addition to the inadequacy of financial resources, other essential materials for work such as vehicles and medicines which are essential in preventive chemotherapy and laboratory investigative materials such as testing kits and chemicals are unavailable.

*“Many a times we face issues of funding in some aspects of our activities*. *I mean the training and drug administration*. *Funds are not enough*. *I will say it’s not the best*”

(Respondent 1, National)

One cause of the inadequacy identified was the delay in release of funding and other essential materials from donor partners for programme implementation. Some respondents added that though activities are being sponsored, funds are sometimes not released on time. This creates a problem where some activities are already carried out with the minimal budget available before money is made available later in the fiscal year. Another cause of this problem was the multiple partners sponsoring a particular activity and the differences in their spending periods. Hence, though a budget is drawn with the proposed funds of all supporting agencies, some are released earlier than others hence leave a deficit in the budgetary allocations.

*“When they bring the funds*, *we do the activities*. *But most times we do not have all the resources we need*. *We can budget for things but they won’t happen because no money*”

(Respondent 11, District)

*“We use different sponsors for budgeting but because they have different spending periods*, *the monies do not come same time*. *So always the money is not enough*”

(Respondent 2, National)

### Dwindling volunteerism

Respondents explained that in order to ensure wide access to services under the programme, the concept of volunteerism was adopted to empower community members to own the programme. However, across all the levels of implementation, it was identified that the utilization of community volunteers in recent times for programme activities has dwindled.

*“Now the volunteerism is going off*, *so they expect to be paid*. *But previously when they understood it as volunteering*, *that time they were even in touch with us*”

(Respondent 12, District)

It was explained that as the economy of the country changed, community members who hitherto willingly decided to volunteer to support implementation of the programme within their communities started to make greater financial demands. This as reported by some respondents was not in line with the concept of volunteerism. A respondent shared the sentiments of volunteers that in the previous years, volunteers were given some priorities in the communities in addition to small remuneration from the programme. However, such financial demands by volunteers have increased which cannot be afforded by the budgetary allocation of the programme. Also, priorities given to volunteers in communities which include exemption from communal labour, free labour on farms among many others have since been non-existent coupled with the inadequacy of logistics and lack of motivation. Hence the withdrawal of many community members from volunteering in the implementation of the programme.

*“Now as the name connotes*, *it is supposed to be voluntarism but often in recent times they don’t see it as voluntarism again*, *they see it as a paid job*. *They are asking for all kinds of things and you can’t blame them*”

(Respondent 4, National)

Respondents noted that this poses a great challenge to the programme since in most endemic communities, the formal health service do not have the adequate number of skilled health workers to carry out all activities especially moving from house to house to ensure that everyone is covered.

*“Some who used to volunteer have very low motivation it’s because they see it as additional work load for them to perform*. *In a way they expect some motivation which doesn’t come*”

(Respondent 11, District)

### Competing health programmes

Further enquiries into the implementation challenges revealed that the presence of other vertically implemented health programmes in the health system was sometimes a challenge to the effective community level implementation of NTDs programme. Some respondents explained that with the existence of programmes such as the National Malaria Control Programme (NMCP), National Aids Control Programme (NACP) which are equally implemented by the same officers at the regional and district levels usually draw more attention and are considered more well-funded programmes. This leads to the neglect of the NTDs programme in most areas until such competing programmes are fully implemented.

*“The way NTDs people come*, *the same way malaria and HIV/AIDS too and we are the same officers*, *so attention is given to those who have the funds available*”

(Respondent 7, Regional)

Respondents added that competing programmes are also a contributory factor to the dwindling volunteerism on the neglected tropical diseases programme since other vertical programmes provide higher remuneration packages. Also, community members clearly see the impact of diseases such as malaria and HIV/AIDS more than the subtle impact of most diseases termed neglected. In terms of amount, a respondent reported that an amount of 100 Ghana cedi was usually given to community-directed distributors (CDDs) who volunteer for a round of mass drug administration which involves multiple whole week visits whiles other programmes provided more to these volunteers.

*“We give about GH₵ 100 for their services but when they compare to what they receive from malaria and HIV which is giving them quite a substantial amount*, *then they think someone is stealing their money*”

(Respondent 2, National)

However, a respondent added that though the other programmes could be competing, usually at the lower levels some funds are diverted from these programmes to the NTDs programme when there is an excess.

### Poor social mobilization

On implementation in the communities, poor social mobilization and ownership of the programme was identified as a major challenge to effective implementation of the NTDs programme. Respondents noted that across all levels, the synergistic power of all social stakeholders has been poor but poorer in the communities. Some respondents added that the mobilization of all influential groups in the community was needed in raising awareness and delivering programme related resources and services to identified and affected communities.

*“There are some challenges that are operational*, *with acceptability of the programme in some areas and mobilization*”

(Respondent 5, National)

This challenge has led to limited awareness on neglected tropical diseases and its impact on affected communities, reduced coverage, poor community ownership and acceptance among some affected communities. Further enquiries revealed lack of awareness, poor community engagement and lack of understanding of the burden of NTDs among communities as some reasons for poor social mobilization. A respondent noted;

*“You see*, *the issue is that the people are not aware of some NTDs or are not aware how it directly affects them*”

(Respondent 13, District)

### Weak governmental/political commitment

Poor commitment from government was cited as a challenge to implementing strategies under the programme and meeting programme objectives. Though the NTDs programme is under the Ghana Health Service which is fully a government agency, respondents especially at the national level revealed that government support only covered salary of officers on the programme and office space. However, all other activities in relation to implementing the programme do not receive any support from the government and purely runs on donors/partners’ support.

*“Lack of governmental support*. *When I say governmental*, *I want to admit that government takes care of our salaries and infrastructure as in our offices but you can see the financial commitment is not there*”

(Respondent 6, National)

This they reported gives the government and technical officers little influence on the key objectives and implementation plan of the programme. Additionally, respondents noted that the absence of political will was a challenging factor. As compared to other diseases reported in the country, neglected tropical diseases receive very little attention from political powers. Most political parties or governments do not consider NTDs as part of their campaign policies as they do for other diseases. Hence the further neglect when such political powers are in control of government resources.

*“If I will add*, *the way the government has been promising so many things during campaign about other programmes*, *NTDs does not have that*. *The big people do not see it as a problem since it does not affect them*”

(Respondent 4, National)

Enquiries about the reasons for weak political commitment revealed that, low level of advocacy and less appreciation of the burden of NTDs especially because it thrives among the poor were major reasons why NTDs do not get adequate political attention.

*“You don’t see people advocating like that*. *Even people don’t know how it affects them so they don’t even worry*”

(Respondent 1, National)

### Weak monitoring and supervision

Respondents stressed the importance of effective monitoring and supervision and noted that, it has been a great challenge to implementation of the NTDs programme. It was added that, in relation to reporting on programme activities from the district, regional to national level, there are usually several disparities in submitted reports. This is due to the weak monitoring usually at the lower levels.

*“One thing is monitoring and supervision and documentation*. *They can send you a report that they have covered something you will go and look back and it doesn’t tally*”

(Respondent 3, National)

A respondent narrated that, sometimes the number of medications given to the community volunteers do not tally with the numbers captured in the reports. In that instance, national officers would have to go down to the community for head counts. Most often issues such as wrong tallying, diversion of programme drugs into private business and mal-handling of medications are detected. Due to these, the programme is unable to account fully for donations received from partners and also deprives some affected areas of programme drugs due to shortage. A respondent indicated that;

*“You will receive reports and realize there is something fishy*. *When you go down*, *then you will see the community volunteers have made mistakes or diverted some drugs*. *That one we let them prepare all the reports again*”

(Respondent 5, National)

Some respondents added that due to the weak supervision and monitoring, it was difficult to identify efficiently whether the activities were actually being implemented as planned, determine which parts of the programme were deficient and needed redesign. However, it is noted that currently monitoring and evaluation on the programme is being improved to ensure effectiveness.

## Discussion

It is essential in every policy implementation process to identify possible and existing challenges in order to adequately meet policy objectives. Challenges of implementation reported in this study were inadequacy of resources, dwindling volunteerism, competing health programmes, poor social mobilization, weak governmental and political commitment and weak monitoring and supervision.

In a recent study on the implementation of the Community Based Health Planning and Services plus (CHPS+) in Ghana [15], health system challenges such as inadequate funding for implementation of health programmes at the community level, lack of essential logistics and lack of community ownership were reported. These findings are in line with the findings of this study which also noted lack of funding from the state and the reduction in donor funding as a result of Ghana being a lower-middle-income country. However, their study further reported issues such as lack of proper community entry, distance to CHPS facilities, lack of security and late reporting of cases. Other challenges that were not in line with this study could be due to the further engagement of the community members in the study on CHPS+ which was absent in this study [15]. Most studies on health interventions in Ghana have also reported inadequate funding and inadequacy of logistics as challenges to effective implementation [16–18]. Despite the differences in context and economic growth, inadequate funding has also been reported in studies in Tanzania [19] and Argentina [20] with management and maintenance of resources also noted as a major cause.

The study in Argentina corroborates the findings of this study that lack of political support was a significant barrier to the implementation of community-based health interventions [20]. Most NTDs do not have acute implications on people and the burden is often among poorest of people in a country, hence most politicians do not appreciate its impact on the economy. Thus, it becomes difficult to get such issues on the political agenda as compared to other infectious and non-infectious diseases of acute nature. A qualitative study among health care professionals which was similar to this study reported challenges such as poor communication and lack of collaboration which were different from findings in this study [21].

In another study, barriers reported include the lack of clear roles and the complexity in navigating through the multiple dimensions of the programme [22]. Although the NTDs programme in Ghana is also multi-dimensional, navigating through the dimensions was not challenging as compared to the community-based child weight programme [22]. According to a scoping review on barriers to implementation of sexual and reproductive health programmes, literacy issues, financial constraints, culture and discomfort about issues of sex were pertinent challenges to implementation [23]. Some of the findings could be due to the concentration of the study on sexual and reproductive health and the sensitive nature of the research area. In terms of dwindling volunteerism, the state of the world’s volunteerism report highlighted the positive impact of volunteerism on the achievement of community-based programmes [24]. Thus, a decline in volunteerism as reported in this study could lead to inefficiency and ineffectiveness. According to some studies on volunteerism [25–27], reasons for decline in volunteerism include increasing volunteers’ expectations and the changes in the way of understanding volunteerism. Similar reasons were noted for the decline in volunteerism on this study. In Ghana, this could also be attributed to the top-down approach of implementation of the programme where interventions emanate from the national programme which creates a mismatch between programme and community priorities. Similarly, some of the diseases (Schistosomiasis and Soil Transmitted Helminthiasis) are catered for by teachers through school distribution programmes where programme medications are distributed in schools [28]. Hence additional community volunteers may not be engaged for such purposes.

A report summarizing possible implementation barriers explained that barriers may be rooted in diverse causes which included opposition from major stakeholders. It concisely reported lack of coordination and collaboration between parties, lack of motivation and political will, conflicts between existing policies, lack of operational guidelines, human and financial resource constraints as some of the challenges that almost all programmes encounter during implementation [26]. In the Ghana Health Service, the main strategies utilized were mapping to determine endemicity and mass drug administration (MDA). Under MDA, drugs are administered to people and communities at risk. Ivermectin is given to communities annually and sometimes biannually for onchocerciasis, with Praziquantel administered for schistosomiasis. Also, Albendazole or Mebendazole are administered for soil transmitted helminths often among children of school going age whiles Ivermectin and Albendazole were given for lymphatic filariasis [2]. The challenges identified in the study largely affect the distribution of these medications, availability and accessibility in the endemic communities. Ensuring that interventions are community-owned, educating people on volunteerism and placing NTDs on the political agenda could to a large extent improve upon the mass drug administration strategy and the programme as a whole.

Several studies that corroborate with findings of this study imply that there are diverse challenges to the successful implementation of programmes within all sectors. Though some of the challenges are evident across multiple sectors, others are specific to the particular policy and programme as evident in this study and other studies reported [15–28].

## Conclusions

Health programmes as open systems are affected by several factors in the internal and external environment that impede the successful implementation of the NTDs programme. As seen in the study, multiple factors work independently and together to impede implementation of the NTDs programme. These challenges when identified creates opportunity for holistic approaches to truncate their effects on the programme. It is evident that though successes have been recorded on the programme, there are still major obstacles in relation to financing, capacity, ownership and accountability that need to be addressed.

### Recommendations for improvement

In the face of reducing donor supports, it is recommended that government increases its ownership of the NTDs programme by going beyond provision of human resource to fully making budgetary allocation for major programme implementation resources to ensure sustainability. A combination of top-down and bottom-up approaches should be adopted in order to ensure full community participation and ownership of interventions and improve volunteerism. There is the need to build capacity of programme officers in monitoring and evaluation on the NTDs programme with an extension to all health interventions to ensure accountability and efficient use of resources to achieve effectiveness. Further research is recommended on the contributions of external stakeholders to the NTDs programme and how it influences their power and control over implementation activities in Ghana.

### Study limitations

This study was limited to the state implementing agency (GHS) without the views of other non-state implementing agencies and community members who could have provided other perspectives to implementation. However, the Ghana Health Service and the national NTDs programme controls major aspects of implementation hence could still provide relevant information.

### Ethical approval and consent to participate

Ethical approval to conduct the study was obtained from the Ghana Health Service Ethics Review committee with certificate number GHS-ERC 065/11/19. Written informed consent was obtained from all participants, Covid-19 protocols were observed during data collection and all ethical research principles were upheld in the study.

## Supporting information

S1 Text Sample of Transcribed Interviews. (PDF)
