# Supplementary material for: Challenges of implementation of the preventive chemotherapy neglected tropical diseases programme in Ghana
Source: PLoS Negl Trop Dis. 2023 Feb 21;17(2):e0011116. doi: 10.1371/journal.pntd.0011116 (PMC9983826; doi:10.1371/journal.pntd.0011116)
Supplement: S1 Text — (PDF) [file pntd.0011116.s001.pdf]

Interview with NTDS focal person at the Ga West Municipal Health Directorate, Amasaman on Implementation of the NTDS programme.

Total time: 25:40mins

Designation: Disease Control Officer/ NTDS Focal person

Q1. First, I would like to know what you know about the NTDs control programme in the district.

ANS:

So, I know the municipality handles a number of the NTD programmes within the municipality. Per the Municipality, any neglected tropical disease you want, you will get when u look into our surveillance data. However, those that ermm, there are available drugs for management, we manage them. Currently when you take like Leprosy, we used to have the only leprosy officer left in Ghana here but he went on retirement in 2018 so after he went on retirement, even though we have learnt under his feet and could manage the cases and everything, I think nationally they decided to mean kind of send the management of leprosy cases from the district to the regional level. So, we stopped managing cases cos we were not being given the drugs and everything. So now all leprosy cases we suspect and those who are on treatment, when they come, we refer them to Adabraka, that's where they are being managed currently.

We also have Yaws, but there is no national programme that is being implemented for its management. So, for those ones when they are identified, they are managed clinically at the facility level. But for Schistosomiasis and Soil Transmitted Helminths, those ones the NTD programme has a programme in place for it. That is the yearly administration of Albendazole and Praziquantel. So errm 2019 programme was held somewhere around November/December and we even continued up to January. We did the School drug distribution for school children in KG2 up to JHS 3. So, for Schistosomiasis, we are doing it. For LF currently we are not doing the community drug distribution again. What we are doing is case management. So, when we identify the LF cases, we educate them on leg hygiene, leg care, management of acute condition, and we give them their annual drug administration. So, every year they need to come back to us for a review. Not to us at

the district level but they go to a facility that is closer to them so for all our public facilities, they do manage LF cases there.

Q2. So, in your opinion, what do you think is the importance of the control of these diseases in the municipality?

ANS

Hmm, you know ermmm the burden that these NTDs bring to people, when you look at it, it is very much important that it is controlled and even prevented among the populace. Because when you meet people who are infected with let’s say LF. When the oedema sets in, when the lymph oedema sets in, they are unable to do their normal work they have to do cos the moment you stress the leg too much it becomes an issue and they get into acute crisis and those things. So, it brings about stigmatization, they become economically dependent on their family members and those things. SO it’s is very much important that such programmes which will prevent the development to such disability stages come in however, the acceptability by the individuals who have to take the medications that is the community members is also an issue cos when we were doing the community drug administration, we know the benefit we communicate it to community members but some refused to take it. So, for me as a Dx control officer, I know the importance of the programme, the drug administration, the benefit one stand to get wen they take it. Yes, still the acceptability because of individual’s perception and misinformation and other things they tend not to take the drug when we take it to them.

Q3. So, you have mentioned some of the efforts the municipality is putting in in the first question, so how would you compare the efforts currently to previous years. Would you say the efforts are now more intensified than previous years?

ANS

I would the efforts to some extent is rather dwindling. Yes, because when we used to manage the leprosy patients here our selves it was very vibrant, surveillance was up high, people were even

referring from other districts to this place. You get it, so we were really up and doing. Connected to all other districts because of cases that they have referred here but now everyone is relaxed because cases are not coming and even when they come, they have stopped coming themselves because they know when they come they wouldn't get their medications, they wouldn't get the sort of help they think they would get. However, for the LF, they come and we are also doing surveillance on them. When we identify them within the communities, we bring them to the facility then we give them the necessary education then we dose them with the drug before we leave them and we are in touch with them. So, for now I will say it is not as vibrant as it used to be.

Again, it is because errm the community volunteers. Now the volunteerism is going off so they expect to be paid. But previously when they understood it as volunteering, that time they were even in touch with us. They will identify the case in the community and they will even call you to come. Now that urgency of calling us or connecting with us in terms of surveillance has died off because they know when they come, they wouldn't even get their transport or anything so that part too.

Q3. So aside the taking away of the management of the cases and the dwindling volunteerism, is there any other factor why it is dwindling in the municipality.

ANS

For now, No. The issue is it is not completely died off, we are on, its surveillance is still ongoing, active and partial surveillance is ongoing in the facilities. They are looking out for the cases and those things but then what I meant by the dwindling is that when a place becomes a centre of something. You realize that all activities and vibrancy centres around that thing. But the moment that thing is removed then the kind of enthusiasm and everything everyone is putting in comes down a bit. So that is what is happening now it looks as if we are not seeing the cases as we used to see so the energies have gone down a bit but then what ever we need to do as surveillance officers in terms of case identification, case management and referral we are doing it.

Q4. In controlling of the NTDs, do you coordinate its implementation with other programmes in the health sector or you do it alone on its own.

ANS

You see management of these NTDs, they are distinct in themselves. Because the drug management is even different. The drug management for LF is different from the drug management for Schistosomiasis. So, for the programme itself you cannot mix it up with other case management so you manage them on their own.

Q5. Is the same way the Disease surveillance, M & E, planning is all done separately?

ANS

So, when it comes to the surveillance, we are operating or let me say Ghana is operating the Integrated diseases Surveillance and response so it is integrated when you take our monthly report form, monthly morbidity form. You report all the diseases you have seen in the facility and it includes the NTDs. So that is the integrated surveillance, so it is not parallel it is integrated just that for some of them, LF, BU and Leprosy, so as part of the monthly reporting it is part of the surveillance we do within the month and we report. We don't do surveillance on them and report parallel. It is integrated so we do it together with all other.

Q6. So, in you district health plan, do you factor in the control of NTDs. So as at this particular year. Do u have NTDs control.

ANS

So, we for the district we have our plan and we factor in NTDs. But for the programme to be implemented, it depends on national. They decide when to implement and NTD programme. So for us we make an adjustment. As and when they want to us to implement it, we make room and we implement it.

Q7. Thank you very much. So far, the ones you have been able to implement successfully, what are the main factors that have helped in the implementation.

ANS

So, for ermm, let's say last year the school-based schistosomiasis programme, it was successful however it has its own challenges. The public perception of the medicine and some adverse events that are recorded. So, though it is successful we have about 70% success rate. I mean getting about 70% of the target population taking the medication but then it comes with its own challenges about public perception about medicine, adverse events following immunization and then low motivation of the teachers, you know it is the teachers who do the drug administration when it comes to the administration when it comes to the school based programme. For the district health directorate, we kind of manage the drugs and distribute them and train them as to how to do it, but the drug administration is done by the teachers. So, some of them have very low motivation it's because they perceive it as an additional work load or an additional duty for them to perform. In a way they expect some motivation which doesn't come.

Q8. These are lots of challenges. So, the 70% success rate you mentioned do you have any factors or reasons why you were able to achieve that success rate. 70% is high so any reasons why you were able to achieve that? Maybe with budget, people were interested etc.

ANS

As for budget, hmm the budget that comes with the programme implementation is for training. Training of the teachers on how to administer the drug. Then there is also a component of fuel for education to do their monitoring in school then for health to do their monitoring in school. Apart from that there is no other remuneration that comes. So it is not about the money, the issue is that the implementation of the school based programme in public schools are very easy and successful because they are government schools and the teachers and head teachers easily buy in, the education directorate has an oversight responsibility over them and they are able to kind of convince them, talk to them and they accept. But our main challenge is with the private schools,

that's where we face a lot of challenges, some of them will come and take the drug and will just return it that the parents said No and those things.

So, for the success of the implementation if the public schools, is the mere fact that they are government institutions and they accept it.

Q9. In this case, what do you think could be done to improve implementation of this particular programme?

ANS

For the implementation of this particular programme, what we really need to do is the public "buy-in". There has to be a lot of education and sensitization about the drug and the programme in the public and there should ermm, the implementation shouldn't be ad-hoc, what we realize is that sometimes we are given very short notice to implement the programme, so as a municipality, we don't even have the ample time to do the communication. Cos in some schools you send the drugs and everything to them then they will say they are sending a consent form to every parent to endorse before they bring. You get to the school and they tell you parents have not et endorsed it. And because the time is short, you go there one, two and you can't go there again or the teachers won't administer and the programme period will be over and we will bring the medications back. But then if there is ample time, so from the beginning of the year, if they know in December or November we are going to implement the programme so we can start the sensitization as early as possible so before it gets to that time, every parent is aware, everyone is aware and the acceptability will be high.

Q10. You know in every programme there are stakeholders, can you identify some of the stakeholders you work with as a municipality?

ANS

When it comes to health, when it comes to Schistosomiasis, our main stakeholders we work with is Ghana Education Service and the school's PTA. So, with GES, when we get them, we get the

teachers because they are the main administrators of the drug. Then the school's PTA so we get the buy in of the parents. We also involve the assembly men because there are also within the communities where the schools are cited and they live with parents and those things so they are also part of the stakeholders we work with.

Q11. Can you identify any advantage or disadvantage or working with any of these people?

ANS

For now, I think having them is more advantageous than a disadvantage because their awareness and involvement in the programme enhances the uptake. It enhances their uptake.

Q12. So, in your own judgement as the one inn charge, how will you assess the progress of the municipality towards the implementation of the programme?

ANS

I am a bit confused about asking of progress because it is not like something which is there that gradually we work to achieve. No, as and when they bring it, we implement it, report it and we implement it as we have to implement it. And for that, we do it to what is expected of use to do. So, train teachers we train, give the drugs we give, monitor we monitor, bring report we bring. So in that case 100% we do what we have to do.

Q13. But with surveillance, the aim is to make sure that all these diseases recede. So, with surveillance is it showing that all these particular problems are receding?

ANS

Yes. Yes. It is showing that it is reducing, however, you know the health seeking behaviours so you cannot really tell because this is a passive surveillance. We are waiting in the facilities as and when they come, we document. Ans you know for passive surveillance you cannot say that is the

true reflection of what is happening in the community. So, per our data we are seeing a decline in numbers but then maybe if we should do an active surveillance or community surveillance, more cases might be identified.

Q14. So, with the resources that are available now, would you say that in case some of these stakeholders withdraw would you say the municipality might be able to continue the control of these diseases?

ANS

It will be difficult because all stakeholders are important here. Taking into consideration like the school-based programme we are doing now for Schistosomiasis and STH, if the teachers withdraw, unless the NTD programme is ready to pay volunteers who will go around. SO, should any of the stakeholders withdraw, then it will be a disaster.

Any other Information?

For now there isn’t much, but you know as the name implies, the diseases themselves are neglected and sometimes you meet patients who are affected by these diseases, you will really know that they need that help and support and some reporting very late when disability has set in and therefore you cannot do much to help them. And the programme itself I think its financing is something else, so all the time, I mean the implementation sometimes doesn’t really go as it is supposed to go. Because at the end of the day you need to receive adequate resources for you to be able to execute the work to the fullest. However, whatever you receive you use it to do whatever it can do. And that is it.

Interviewer: I am very grateful for your time.

Interview with National NTDS Monitoring and Evaluation Officer on Implementation of NTDS Programme

Q1. Please describe what you know about the NTD control programme in Ghana.

ANS

The NTDs programme is a programme which started in Ghana for the PC, that is preventive Chemotherapy and started implementation somewhere 1974 with control of the Flies that is the Black fly control under the APOK and from there, Ghana did a remapping of the disease in country from 1998-2000. For Onchocerciasis we did a remapping in 2008, Schistosomiasis, STH we did the remapping in 2007, 2008. For LF we did in the year 2000.

So, what we normally do is to administer the drugs to endemic districts. That is under the PC. So, our main intervention is in 3 strategies. We do Mass Drug Administration, Health Educations and morbidity management and control. We have the CM that is Case Management and they also have their programme managers. They search for the case and manage it but we do MDA to endemic districts. That's the Overview of NTDs

Q2. In your opinion, what importance does the control of these diseases have in our health system?

ANS

Owww Okay. The burden of these diseases in country is very high. IT affects the poor so it has influence on the economy, if somebody is poor and the person has the NTDs, the person becomes poorer and poorer. So, impact on the socioeconomic, the culture, the growth of the economy all, NTDs contribute to these factors but it does not affect the rich so we think it is being neglected.

Q3: What are your top priorities in terms of NTDs control?

ANS

So, under the PC, we are dealing with 5 diseases. Onchocerciasis, Elephantiasis, Bilharzia, Soil Transmitted Helminthiasis and Trachoma. But our main priority is to eliminate all these diseases. So now we as a programme mut move from control to elimination. Trachoma has been eliminated in 2018. LF we started with 98 endemic districts and we have been able to break transmission in 83 districts so its left with only 15 more to go. STH and Schistosomiasis is still under control. But from our impact assessment from 2007/2008 and the impact we did in 2015 tells us that our prevalence of Schistosomiasis and STH can be eliminated in Ghana. So, our target for elimination is 2025. If the government brings all these factors: environmental factor, personal hygiene and the WASH into it we know we can eliminate Schistosomiasis and STH by 2025.

Q4. So as a National programme officer, can you tell us some of the current efforts being put in place within the health system to control these diseases.

ANS

Because these diseases have been neglected, once thing the programme is targeting is to now not let it be neglected again. Because we are attending to it. What we are doing now is to include these NTDs and its indicators into the health information systems. That is the DHIMS and now its being integrated. Now we are asking the NMC to include the NTDs into their curriculum. We also talking to the government to support the NTDs programme after partners have gone out. We are developing a document, we have now finished with the draft and sent to the Ministry.

Q5. How will you compare the current efforts to the previous years?

ANS

Oww there has been a great change. At first what we only do is case control we only do the drug administration but not thinking about including into the GHS indicators that they use to rank directors and officers. HIV and Malaria is part of the indicators that they used to rank regional and district health directors but now NTDs is included.

Q6. Can you please give us some of the factors that have led to the great change you talked about?

ANS

At first because it was under control, most of the times it was just MDA but now we want it to be integrated into the GHS streamline. So now when you go online the DHIMS now you can see NTDs. This is first of its kind. And now what we are doing is to train nurses on how to manage the lymph oedemas that is the Elephantiasis. AT first if someone has LF and is in Tamale, the person must move from Tamale to the national office for management because of lack of capacity building at the lower level. So now we have put it as a mandate that if malaria case can be managed at the CHPS compound, then lymph oedemas too should be managed at the CHPS compound.

Greater Accra Regional

Q1. Can you describe what you know about NTDs in the Greater Accra Region?

ANS: Like I told you, we Greater Accra, we are not affected by most of them. I know of only Elephantiasis. Even that has been phased off because of the intensive Mass Drug Administration. And even with that, Greater Accra most of the diseases that you mentioned, the 5 or even 10. You know because we are in the center and people come in and out, there is likelihood that there will that there will be that transmission. And because we form border with most of the areas for example Eastern region where they deal with most of the NTDS like Oncho and the rest and you know the Blackfly which transmits the Oncho can travel up to 300km in search for food. SO it goes and stays. So they cannot say they will not treat. ……………………………………………….

Q2. What do you think is the importance of controlling these diseases?

ANS: It is very important because like I mentioned because we share border we cannot say because they mapped out and didn't see anything they will not include Greater Accra. We did it but subsequently they needed to follow up to examine whether it was beneficial to the region.

Other than that it will be like there is nothing wrong with you but we are treating you. It was like an abuse of the medicine itself which we didn't buy, it was donor given.

For the deworming, we are dealing with worms and it is not only intestinal worms, we also deal with Schisto/ Bilharzia and the drug that is given can be used for both intestinal and urinary Schisto. Most of our children you know the volta lake area and even in some areas because they swim, most of them report of urinary Schisto. Bearing those ideas that it's a sign of maturity, everybody would have to pass blood in urine so we take advantage to treat them all. It is mostly school-based.

Q3: How will you compare the current efforts to previous years?

ANS: owww, you see now things are moving on. Things are becoming dynamic more IT or online so things are changing. Those days they tried but it was sought of some crude techniques being used. But these days cos of electronic system things are now simpler and we can easily access the drugs anytime you want. Formerly it was like some catchment area but like I began if a certain area is demarcated for a particular thing it doesn't necessarily mean other areas are excluded. We have areas like GA south, GA West, GA North. They are typical rural and they are in greater Accra. There is a forest area when you come to Ga East, Abokobi. And we cannot say we will not treat.

The only challenge with the STH is that, at times you will give a clear instructution that take this drug which we know is safe, eat before you come. Because if I am treating our child free of charge, for you the parent to also help, they want to leave everything on you that you would have to feed them. And we take advantage of the school feeding programme. We come and find out when the children will eat so that after eating we do the administration of the drug. And because it is school based, we deal with the teachers. They coordinate all the activities. The only challenge is somebody will not eat and there are some conincidences. The just ended recent one, two people died, it was a coincidence in Shai Osudoku. For that matter it affected subsequent programmes whether the school deworming or any other programme which Is health wise.

Q4. Please in relation the health system at the regional levels. Do you capture NTDs in the regional disease control plan?

You know we have the plans but the funding is not there. SO you cnnot carry it out. Even these days the are trying to put all NTDs together for example leprosy, Buruli ulcer because they are all skin conditions. And the recent launch. Say no to NTDs, all these programmes they were there.SO now it's one entity. There is no difference between this and that. These days funding I becoming a problem and we want to say goodbye to verticalization of a programme. Bacause if it is a vertical

programme, the question I ask myself is how do I sustain the programme.cos if you pump money into it for about 3 years and you withdraw then it means the programme has to collapse.

Q5. In relation to integration of the programme, how will you rate the integration.

If it is integrated, people are not recognized cos they see it as neglected. How do I come here.

Q6. What are the main factors that have helped implementation so far?

Oww it is the collaboration with GES. We do not do it in isolation. Infact even with the STH, it is sch based and cos drugs are involved that why the health comes in. and because it is a neglected tropical disease, and the are from health. They cannot write for example cheque to the GES.So we train them on how to administer the drugs and if there is anything they have to inform us.

Q6. What are some of the challenges?

Challenge is a key challenge.

Interview with NTDs focal person at the Ga West Municipal Health Directorate, Amansaman on Implemention of the NTD Programme.

Total time: 31: 54 mins

Designation: Programme Officer /NTDs Focal Person

Q1: So we will just like to know what you know about NTDs, neglected tropical diseases in Ghana?

Ans: So when we talk about NTDs, NTDs we are talking about, NTDs is an abbreviation it stands for neglected tropical diseases right. So, we are talking about a group of infectious diseases that are ……. about 1billion people worldwide, they normally thrive in places of impoverishment, poor sanitation, poor access to water and then in places where there are urban slums and in conflict zones, these are the areas you normally find NTDs and currently there are about 20 NTDs worldwide. It used to be 17 but 3 have been added on recently by the WHO. We have scabies that have been added on, snakebite and one other, I will check and inform you. But about 20 NTDs currently but in Ghana we don't have all the 20, yes. We have PCNTDs, PC stands for preventive chemotherapy and then we have the CMNTDs, the CMNTDs stands for the case management so you find the cases and you manage them. So we are talking about buruli ulcer, yaws and the like. And what we do here is the PC, preventive chemotherapy. So you give drugs to control, you do health education, you do assessment to see where we have gotten to. So there are 5 conditions that

we manage here. Schistosomiasis (solid transmitted hermens), we have trachoma, we have onchocerciasis then we have lymphatic filariasis that's what we call elephantiasis but fortunately trachoma has been eliminated as a public health concern and that was in August, 2018. WHO certified Ghana as the first country sub-Saharan Africa to have eliminated trachoma. So these are the NTDs as we have them in Ghana and elsewhere.

Q2 So please in your opinion what importance does the control of these diseases have within the health system?

Ans: You see as I keep saying to people, these diseases are neglected and people don't pay attention to them but they keep people in a cycle of poverty. I will give you a few examples if somebody is affected with lymphatic filariasis, elephantiasis you are unable to go out to work so you remain indoors, you are unable to farm, you are unable to do anything. Your source of economic income is cut off and it keeps the family in a cycle of poverty and we have something we call DALYs, DALYs is an abbreviation DALYs Disability Adjusted life years the one posed by the NTDs if you put it side by side to what is posed by the malaria, the TB, the HIV/AIDS, it comes nowhere near the NTDs but because these ones have been neglected in terms of budgeting and public health importance, people don't pay attention to them but they keep people in the cycle of poverty and it normally affects the poor people and that is where if you should ask me, that is where are attention should go as a people, right.

Q3 So currently can you give us some of the efforts that are been put in place to control………..

Ans: As a programme as I said our mandate is preventive chemotherapy we try and prevent the condition before the even escalate so we do treatment at the community level and then we also do treatment at the school level. So we do school-based deworming exercise were we partner with our colleagues from the Ghana Education Service. They have a unit called SHEP, School Health Education Programme, yes, so we partner with our colleagues from the Education Service and then we do annual deworming of all school children and we also go to the community, the endemic communities but before then we have done what we call baseline studies so we are aware where the conditions thrive in all over the country. So if you take onchocerciasis I can tell you that in Ghana, its only Greater Accra that is non endemic all the other regions are endemic. Elephantiasis it is only Volta and Ashanti that are non-endemic by certain rules, but then all the other regions are endemic so we know where the diseases are and we know the target areas so we go there with the support of our partners and the ………….. domestic programme (MEC) where they give us the medicines through the WHO then we treat people, so we do mass treatment , that's what we call mass drug administration (MDA). The WHO prescribes that if you go to an endemic community, one way of controlling the disease is to treat the people at mass. So ideally, we should have screened to see who is infected and you treat. Assuming you treat Greater Accra and you want to scree everybody in Greater Accra, its going to be an awkward task, very very big task. So

you treat the people at mass it provides immunity for everybody and then you are able to control the disease.

Q3. So please how would you compare current efforts to previous years efforts, would you say you are doing better than the previous years or its coming down?

Ans: oh I think we have done very well as a programme because when you take something like elephantiasis, I'm talking about the old districts now the new demarcations and the number of districts have gotten out of hand and we are unable to keep at pace with what the politicians are doing but then previously the old districts we had about 98 endemic districts of elephantiasis we have been able to control the disease in about 83 of them. So currently we have the disease in 15 hotspots, hotspots mean that these people have treated for sometime and they are unable to bring the level of infection below a certain threshold, yes, but then even there, I can tell you that we do an assessment, 3 more have passed recently so we have about 12 so beginning from about 98 districts we have as low as about 12 district now. It tells you that now there is a lot of efforts. Previously, we had a lot of challenges for instances people really didn't understand the essence of what was being done , you go to the communities that's the challenge with public health, you see a problem you want to solve, the other person doesn't see it. The person is walking very healthy you say come and take medication, why should I take medication, that is the challenge right. So there was a lot of misunderstanding and then for elephantiasis like this we were doing something we call night blood survey, the worm comes in between the bloodstream. You see if you have treated people for sometime you want to be able to know whether you have brought infections to a certain level below the threshold because you cant continue treating people forever. So for elephantiasis one way of knowing whether the infections have gone below a certain the threshold, at a point in time you should stop treatment, you want to assess treatment, do a survey and find out, has the treatment been effective. The worm comes into the bloodstream between 11pm and about 2am. So if you want to see the worm, you go into the communities between that time to take blood samples. And often times we have been attacked. We are coming to take blood samples you come in the night, are you a witch or a wizard. People misunderstood what was being done but we were doing what we call community entry. You just don't walk in, you meet the chiefs, the leaders, the opinion leaders, you explain in details to them what you have come to do, then they tell you we will meet in probably the Roman catholic hall at 5pm. 5pm you set up, you show them documentary, you explain issues, engage the community that late hour about 11, ask them all the questions, allow them to ask all the question they may want to ask and you think that they have understood, the moment you start taking samples somebody will jump from the bush, herh this people they are doing sakawa. This were the challenges we had in the past but currently WHO has been able to and CDC Centre for Disease Control in ……… Georgia, has given us some test kits that is antibody based so you don't have to go the communities at night again, can be done during the day, your finger stick and then. So a lot of efforts have been done in the past but current methods more robust and then more reliable and more sensitive and its giving a much better

security to the staff than it used to do. And now I think our efforts are bearing fruits for people to see so current methods are working for us as a country.

Q 4: So please quickly in relation to the NTDs how would you describe the coordination between the NTDs and other health programmes?

Ans: yeah its important, you see our recent planning meeting there was a lot of discussion on that, we need to argument the efforts of each other. I will cite the example of Togo, Togo has been able to eliminate elephantiasis, they didn't just do it by giving mass drug administration, you the vector for transmitting elephantiasis is the mosquito so they did a lot of bed net distributions and bed net distribution being done by malaria programme just adjacent our office here. So there is a need for us to work together so as we do the mass drug administration, malaria will also goes along with the bed net distribution so that joint effort we control elephantiasis in the country.

Q4B But is it there now or you are forging towards it?

Ans: oh so there are places where we work together but we haven't had this joint operation before and I think the public health system is addressing that, this is something that should be addressed at the very top of the Ghana Health Service because we are all under Public Health, so the director general, the director of public health instructs or they come together so you guys see how you can work together, I think its better. It cuts resources down because if a team is going to do mass drug administration and another team is going to do bed net distribution, they can go in the same vehicle, cut down fuel cost and everything and that is economically much more sensible.

Q5. So please in terms of treatment of other diseases, the integration well, is there any where were if you are treating NTDs you have to go through a normal health system or it's a separate place where you have to go through to be treated, is there anything like that?

Ans: no, what we do is we work through the Ghana Health Service System, yes that's what we do so from here you are suppose to go through the region, then the district and then to the sub district and then you go through the system and there is the provision of the volunteers that we use normally, its done by the sub-district level when we go to the former head system, you cant run a poer system vertical system, no you will have challenges, yeah you go to a community to work and they don't know you and that the acceptance and recognition is not there. You cant just sit in a car drive to a community and say come and take medicine herh, if you are bring poison to them how will they know .

Q6 so please can you identify some of the factors that have helped the implementation of the programme in Ghana?

Ans: right, one of the factors I must say is that we have a reliable health structure in the country, a very reliable health system structure so that if I want to go to lets say the northern region to work, i just don't get up and walk to the northern region, you write a memo here, to the director of public

health, he signs it, it goes to the director general, he issues a letter to the region so the region expects a team from the NTD office to come to the northern region to work. They will also write letters to the affected districts that a team will be coming to do ABCD kindly give them your support so you go and you meet the district director and his staff who are ready to work with you and for me that is what makes the work successful and then the communities themselves if you are able to interact with them in such a way that they own the programme, it is for us, it is about us, not the man who sat in the vehicle from Accra and came , I mean you can take the horses to the river but you can't force the horses to drink. So if you say take the medication and they say we won't take it, what else can you do. So the acceptance in the community, they knowing that we have such a challenge and these guys have just come to support us, they owning the programme and leading the way makes the work much more easier.

Q7 So what about the challenges you face in implementing?

Ans: as for the challenges a myriad of them you see I have been talking about us using volunteers. If you are going to cover lets five (5) districts in a region, these 5 district s you maybe talking about probably 32 communities thereabout. Every community has a certain population size, you can't use only health staff, the work will be too much for them so we have volunteers, the one we call community based drug distributors (CBDDs). They are people who have been selected by the community, they are taken through training and told exactly what to do. So if you go to every community there have these volunteers, some people call them surveillance volunteers and a whole lot of other names but they have been trained to administer drugs and you know the safety profile of the drugs enables non-medical personnel to administer them right. All they need is their dosing pole to be able to measure the height of the person to be able to know the person must take 2 tablets and then they are being supervised by the health staff, so that they don't overdose or under dose people. Now these volunteers as the name connotes, its suppose to be voluntarism but often times and recent times we don't see it as voluntarism again, we see it as a paid job. They are asking for all kinds of things and you can't blame them. You see those days when we started the community directed treatment with ……….. one of the reasons the behind the selection of the CBDDs these volunteers was that, it's the community that have selected them so he is a exempted from communal labour, he gets people to support him on his farm , so if he volunteers his time to help the community, because their treatment is not the static point distribution as other programme do. They are suppose to move from house to house combing the whole community, registering people in the households and treating them. What do they have in return? Now these days can you go and tell somebody Im the volunteer come and help me on my farm, all the priviledges they were suppose to enjoy doesn't exist any longer. So they are asking for some kind of remuneration and we can only give a little allowance. Oh please use this to buy water as you move round so take this to buy a bottle of coke whiles you move round and they think its inadequate and I agree with them, where is the money coming from, there is no money so volunteer allowance and the things they work with, some places they will need a bicycle, because it's a hamlet, houses are dotted far from each other, if you get a motorbike all the best, they need wellington boots and other kinds of things

but the programme doesn't have money. We ourselves, when we are going out to 10 regions at a goal, we need 10 vehicles, the programme doesn't have that number so you have to go and borrow from other programme. So you go to the other programme and they are also using their vehicles what do you and then sometimes we have conflicting priorities. Now we go to the communities to work, remember I said we work through the health system so if malaria has brought a programme , EPIR has brought a programme, NACP has brought a programme, TB has brought a programme which ones come first. Because we are using the same health style so these are some of our challenges. Right, the challenges they are many and then sometimes the money that comes from our partners we have signed an agreement, MOU is a partner, we are suppose to receive this march, within this period to execute this activity, and there are delays so by the time the money is released to you and you get to the community level, there is another programme being implemented by the district, what do you do. Oh we are doing mosquito net distribution at this time, meanwhile the partners are also waiting for their report, what do you do. So if the Ghana government raise money to support the NTDs we appreciate very much what the government is doing. I mean our office space, our salaries but apart from this almost all the money we use for our work comes from partners. The day that the partners will decide that we are walking out, what will we do.

Interviewer: its actually part of my questions, thank you very much

Q8. So if in case I want to know, you've mentioned some of the partners and stakeholders. I have talked to other people they have mentioned a few. I have heard about the USAID, the ASK, the AIM and also the SightSavers. Are there any other partners maybe we haven't mentioned but you know that you work with.

Ans: Oh I think these are the one we have now, as for personally we get support from the WHO, that one is technical. WHO will not give you, they don't have monies for ……… they provide technical support, then we also have Liverpool, they have a unit called CNTD they also support sometimes and then occasionally we get support from CDC, CDC also just like WHO also provide technical support but not any financial support.

Q8B: but Liverpool also grants some financial support sometimes?

Ans: its for the programme, right like when we were doing, I was talking about assessment. So we did this survey in greater Accra we wanted to know how far we have been able to sustain the gains as far a elimination of elephantiasis is concerned so when you go and do the assessment, you realize that prevalence has fallen below a certain threshold which will give you time about 5 years to see if there will be no ……………… or no …………… how would you see and monitor that, that is what we call post treatment surveillance. And so for the post treatment surveillance in Accra, Liverpool CNTD supported. CDC supported the one in Ashanti and then Western but at a point in time, they broke off and then left because they felt that their monies

Q9 Okay, so like you've said, all these people is there any advantages so far you have seen or experience working with these stakeholders, also any disadvantages working with them?

Ans: One of the disadvantage is that, well there are different partners with different interest so you generate report. There are different things SightSavers are looking for in the report, there are certain things USAID will be looking for in his report, there are certain things probably this other partner will be looking for in his report. So for the same activity, you will have to produce three different reports and because they have different interest, its not possible to malgate them, put them together but if you want to put them all together, one simple report from the same activity should kill it, but then they also have different fiscal years. And in Africa, their fiscal year starts from I think September to about the following year March. SightSavers also starts from probably April to another time. so whiles you are looking at Fiscal year17 here, you are looking at fiscal year 20 here for this person and their monies comes different times. It makes planning difficult, it makes execution difficult. One advantage is that at least we have financial support to carry out your activities. We have financial support because without them we would have been able to do. talking about partners I think they left out VRA, I think VRA has been very helpful because of the ………… of the Volta lake they are interested in schistosomiasis.

Q9. So please, so far how will you assess the progress of the NTD program completely?

Ans: I think we have made a lot of thrives and a lot of improvement and fortunately I think that the world is becoming much more aware of the NTDs. Previously, the attention was minimal so the most recent thing that happened was that, you know we have world TB day, world malaria day, we have this this this, the very first time 30th January was set to celebrate world NTD day and this years celebration was at Bankye Abona, for the very first time you see the governing body for anything about health is becoming increasing aware of NTDs. For me that is a good thing.

Q10. So please like you were saying earlier on, currently are there any efforts in place to incase all these partners withdraw their support is there any effort to sustain the programme?

Ans: Yes yes yes yes, Im happy you are using the word effort, a lot of efforts are being made right, there is a unit under PPME right, they have been mandated to solicit for financial support for the programme.

Right, we have had some successes in the past, the then UNIBANK, they came on board and supported two regions, Upper East and Upper West with mobility management of LFAK 6, and then the bank had a challenge. So that is a way of looking internally to generate funds, solicit for support for our programme. So that unit under PPME they are suppose to champion that, where we could have support from the giant pharmaceutical companies in the country, the TELCOS, the mining companies, if they could put a little money bit of money into what we do, probably our programme will become more sustainable, because you cant guarantee the presence of these partners in the country forever.

Q10B: So if they leave today, there will be something to sustain the programme?

Ans: immediately, I wouldn't say we have money sitting somewhere but we believe that the efforts being made will cushion us before eventually we get to that end right

Q11. So in terms of budgeting as a programme officer is there any, aside the external stakeholders is there any Government budget for implementation of the programme, you mentioned salaries and office ..

Ans: that one is for every government employee that one you are budgeted for but what I'm saying is that every year Public Health, Ghana Health Service get its chunk of support from the government. So definitely there is a budget for public health but the programmes under Public Health are many and there are less endowed programmes so it doesn't really ……….. very much to us. But if you have some support from partners and the others don't have anything at all then probably you are being too greedy. But currently we don't have any support like that.

Q12 is there any other comment or any other thing you would wish to just talk about, recommendation?

Ans: I think the NTDs are diseases that have really not gained so much attention in the country and often times I tell people, we have to make a case for ourselves so if anybody in our own small way can be able to create that awareness, advocacy for support for the NTDs, these are not very difficult to eliminate, they are not difficult to eliminate at all as compared to HIV and others, so we just a little support, im sure we will be able to eliminate the NTDs and people will be better of right.
